# Role of Spleen Stiffness Measurements with 2D Shear-Wave Elastography for Esophageal Varices in Patients with Compensated Advanced Chronic Liver Disease

**DOI:** 10.3390/diagnostics15060674

**Published:** 2025-03-10

**Authors:** Cristina Muzica, Sorina Diaconu, Sebastian Zenovia, Laura Huiban, Carol Stanciu, Horia Minea, Irina Girleanu, Mihaela Muset, Tudor Cuciureanu, Stefan Chiriac, Ana-Maria Singeap, Camelia Cojocariu, Anca Trifan

**Affiliations:** 1 Department of Gastroenterology, Faculty of Medicine, “Grigore T. Popa” University of Medicine and Pharmacy, 700115 Iasi, Romania; lungu.christina@yahoo.com (C.M.); sebastianzenovia20@gmail.com (S.Z.); stanciucarol@yahoo.com (C.S.); horia.minea@yahoo.com (H.M.); gilda_iri25@yahoo.com (I.G.); l_muset@yahoo.com (M.M.); drcuciureanutudor@gmail.com (T.C.); stefannchiriac@yahoo.com (S.C.); anamaria.singeap@yahoo.com (A.-M.S.); cameliacojocariu@yahoo.com (C.C.); ancatrifan@yahoo.com (A.T.); 2 Institute of Gastroenterology and Hepatology, “St. Spiridon” University Hospital, 700111 Iasi, Romania; 3 Emergency University Hospital Bucharest, 050098 Bucharest, Romania; 4 Department of Gastroenterology, Faculty of Medicine, “Carol Davila” University of Medicine and Pharmacy, 050474 Bucharest, Romania

**Keywords:** spleen stiffness measurement, 2D shear-wave elastography, esophageal varices, compensated advanced chronic liver disease

## Abstract

**Background/Objectives**: Esophageal varices (EVs) represent an important portal hypertension complication in patients with compensated advanced chronic liver disease (cACLD). Although upper gastrointestinal endoscopy is currently the gold standard for EV diagnosis, recent guidelines recommend non-invasive approaches to assess EV risk in cACLD patients to reduce unnecessary endoscopies. Our study aims to evaluate spleen stiffness measurement (SSM) using 2D shear-wave elastography (2D-SWE) as a non-invasive predictor of EV presence and severity in patients with cACLD. **Methods:** We included 73 cACLD patients referred to our liver clinic over one year. SSM and liver stiffness measurement (LSM) were performed using 2D-SWE, with specific cut-off values applied to rule in or rule out clinically significant portal hypertension (CSPH) according to Baveno VII consensus criteria. Upper gastrointestinal endoscopy was performed in all patients to confirm EV presence and grade. **Results:** Among all patients, 49.3% had no EV, while 50.7% presented with different EV grades (15.1% grade I, 13.7% grade II, 9.6% grade III, and 12.3% grade IV). A strong correlation was observed between elevated SSM values and EV presence, with SSM values > 32.8 kPa highly suggestive of EV (AUROC = 0.95, 95% CI: 0.909–0.995, *p* < 0.001). SSM values exceeding 40.4 kPa were associated with more advanced EV grades. Combining LSM and SSM improved diagnostic accuracy, effectively stratifying EV risk without invasive procedures. **Conclusions:** SSM via 2D-SWE is a promising, non-invasive tool for EV prediction in cACLD, aligning with Baveno VII recommendations to minimize unnecessary endoscopies. Further validation is required to refine diagnostic thresholds and expand applicability across different chronic liver disease etiologies.

## 1. Introduction

According to the Baveno VII consensus, compensated advanced chronic liver disease (cACLD) encompasses patients with significant fibrosis or early-stage cirrhosis who remain asymptomatic, without complications like ascites, variceal bleeding, or hepatic encephalopathy [1]. This classification aims to improve the work-up diagnosis strategies and assessment of liver diseases, with a high emphasis on non-invasive methods that estimate fibrosis progression and portal hypertension risk [2,3]. Identifying clinically significant portal hypertension (CSPH) is essential in cACLD, as CSPH is a major predictor of complications and has high implications for prognosis and treatment [4,5]. Although the hepatic venous pressure gradient (HVPG) remains the most accurate diagnostic method for CSPH, its invasive nature and limited accessibility led to the need to develop non-invasive alternative tools. The Baveno VII criteria suggest liver stiffness measurement (LSM) via transient elastography (TE) as a key non-invasive method, with a cut-off value > 25 kPa to confirm CSPH and ≤15 kPa with platelet count ≥ 150 × 10^9^/L to rule out CSPH [1]. However, several challenges like limited data, lack of validation, and a broad indeterminate range remain. More importantly, there are scarce data regarding the cut-off values in different etiologies of liver disease.

Esophageal varices (EVs), an important complication of portal hypertension in cACLD, require timely identification to prevent bleeding, a significant cause of morbidity and mortality in these patients [6]. Although esophagogastroduodenoscopy (EGD) is the standard diagnostic method, its invasiveness and costs have led to increasing interest in non-invasive diagnostic strategies. The Baveno VII consensus promotes non-invasive screening to limit unnecessary endoscopies in low-risk patients [7].

Spleen stiffness measurement (SSM) using 2D shear-wave elastography (2D-SWE) has shown promise as a non-invasive method for evaluating portal hypertension severity [8,9,10,11,12,13,14,15]. Combining LSM with SSM may enhance predictions of CSPH and the likelihood of varices needing treatment (VNT) [16].

This study assesses the utility of SSM via 2D-SWE for predicting esophageal varices in cACLD patients, in alignment with Baveno VII recommendations. Our findings aim to support more efficient patient stratification, minimizing invasive procedures and improving clinical outcomes.

## 2. Materials and Methods

### 2.1. Study Population

Patients aged from 18 to 75 years who were referred to our outpatient liver clinic with compensated advanced liver disease (cACLD) from 1 June 2023 to 1 June 2024 were considered eligible for inclusion in the study regardless of the etiology of liver disease. The diagnosis of cACLD was based on clinical, laboratory findings, and imaging studies or on liver histology. All patients had LSM by 2D-SWE ≥ 10 kPa. We excluded patients with splenectomy, hepatocellular carcinoma (HCC), porto-splenic vein thrombosis, history of transjugular intrahepatic portosystemic shunt (TIPS), and alcoholic hepatitis.

The study protocol was approved by the Ethics Committee of Grigore T. Popa University of Medicine and Pharmacy of Iasi. A written consent was obtained from each patient with respect to the 1975 Declaration of Helsinki.

### 2.2. Clinical and Laboratory Data

Routine blood tests, including platelet count, prothrombin time, serum albumin, serum creatinine, international normalized ratio (INR), serum aspartate aminotransferase (AST), alanine aminotransferase (ALT), and bilirubin were measured.

The severity of liver disease was determined by the Child–Pugh score and the model for end-stage liver disease (MELD).

### 2.3. Endoscopy

A screening EGD was performed in all patients up to 60 min after 2D-SWE using a flexible EVIS EXERA video gastroscope (Olympus Europa Medical Systems, Hamburg, Germany). Grading of EVs was performed in concordance with the Paquet classification [17].

### 2.4. Two-Dimensional SWE

LSM and SSM by 2D-SWE were evaluated in all patients using the Aixplorer MACH 30 ultrasound system (Supersonic Imagine S.A., Aix-en-Provence, France) with an abdominal 3.5 MHz curved array probe. LSM was assessed on the right lobe of the liver through the intercostal spaces with the patient in the supine position and the right arm maximally abducted during breath hold. The region of interest (ROI) for 2D-SWE was placed in an area without large vessels and bile ducts, avoiding the liver capsule. SSM was performed in the supine position with the left arm in maximum abduction and by placing the probe in the left intercostal spaces. ROI was placed between the central region and the lower pole, in a position near the abdominal wall, via an intercostal approach. The ultrasound examination also included the measurements of the portal vein flow velocity (PVFV), hepatic artery resistance index (HARI), splenic artery resistance index (SARI), and portal hypertension index (PHI) as indirect ultrasound markers of chronic liver damage. HARI and SARI were calculated using the same formula according to hepatic or splenic artery examination: [peak systolic velocity (V max)—end-diastolic velocity (V min)]/peak systolic velocity (V max), while PHI was calculated using the following equation: [(HARI × 0.69) × (SARI × 0.89)] portal vein mean velocity (V mean) [18].

### 2.5. Statistical Analysis

Statistical analyses were conducted using SPSS software Version 22.0 (SPSS Inc., Chicago, IL, USA). For quantitative variables, comparisons were performed with either the Student’s *t*-test or the Mann–Whitney test, depending on whether the data followed a normal distribution or not. Qualitative variables were analyzed using either the Chi-square test or Fisher’s exact test, based on suitability. Relationships among parameters were evaluated using Pearson’s correlation coefficient (r), while differences in ultrasound parameters by esophageal varices grades were assessed with One-Way ANOVA. The area under the receiver operating characteristic (AUROC) curve was calculated to evaluate the predictive power, sensitivity, and specificity for SSM. AUROC values were reported with 95% confidence intervals, with diagnostic accuracy deemed poor for a c-statistic greater than 0.85. The AUROC curve’s optimal cut-off was identified as the point yielding the highest combined sensitivity and specificity. All tests were two-tailed, and statistical significance was set at *p*-values below 0.05. Hazard ratio (HR) was performed to assess the risk associated with the presence and severity of esophageal varices. This model allows for estimating the relationship between increasing SSM values and the risk of higher variceal grades while accounting for multiple confounding variables. Spleen stiffness measurement (SSM) was conducted using 2D-SWE (Aixplorer Supersonic Imagine Mach 30, Provence, France), applying cut-off values from the Baveno VII criteria to confirm or exclude CSPH, with SSM values under 21 kPa excluding CSPH and values above 50 kPa confirming it.

## 3. Results

Among the 73 patients included in the study, the main cause of liver disease was chronic alcohol consumption (53.4%) followed by chronic hepatitis C (27.4%). Most patients had class A Child–Pugh–Turcotte (70%). None of the patients had a history of decompensated disease (presence of ascites, hepatic encephalopathy, or history of variceal bleeding). The upper gastrointestinal endoscopy showed that 49.3% of them did not present with EVs, while 15.1% had EV grade 1, 13.7% grade 2, 9.6% grade 3, and 12.3% grade 4. The mean spleen circumference was 85.05 ± 16.2 mm (Table 1).

Table 2 shows that LSM, SSM, PHI, and spleen size are significantly correlated with EV grades (*p* < 0.001). The increase in the spleen viscosity index (ViPLUS) paralleled higher EV grades, rising from 1.70 ± 0.23 Pa·s in those without EV to 2.65 ± 0.90 Pa·s in grade IV EV (*p* < 0.001).

The subgroup analysis between patients with and without EVs showed significant differences regarding liver stiffness (21.71 ± 6.25 kPa vs. 16.81 ± 1.33 kPa, *p* < 0.001) and spleen stiffness (46.04 ± 12.46 kPa vs. 29.52 ± 3.98 kPa) (Table 3). Similar findings were oberved when comparing PHI, portal vein diameter, spleen size, and splenic ViPlus in patients with EVs versus those without. On the other hand, there were no major differences regarding the arterial resistance indices between patients with and without EVs (HARI 0.71 ± 0.032 vs. 0.66 ± 0.064, *p* = 0.326; SARI 0.74 ± 0.0029 vs. 0.64 ± 0.059, *p* = 0.263).

**Figure 1:**
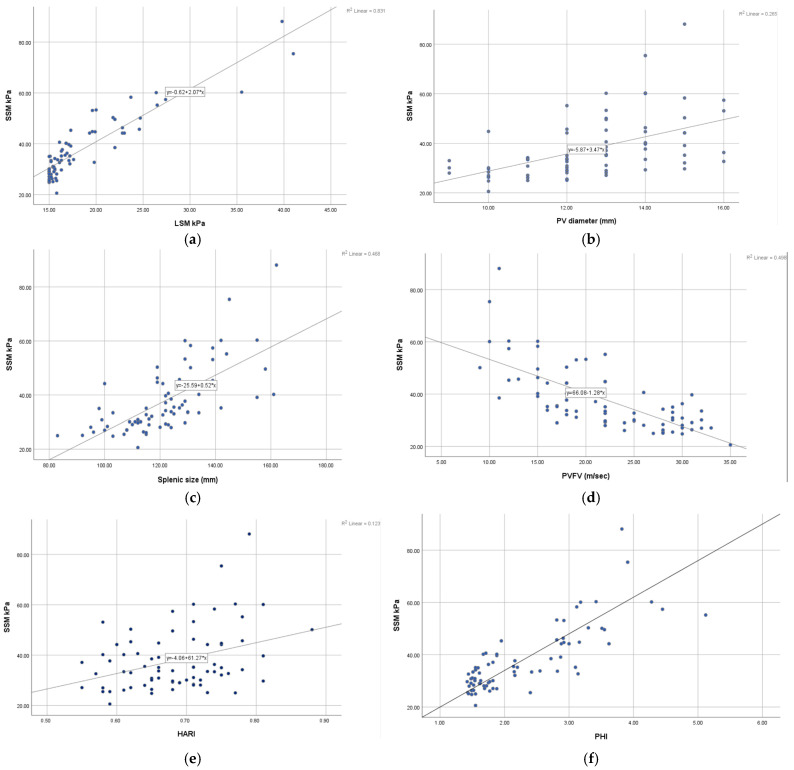

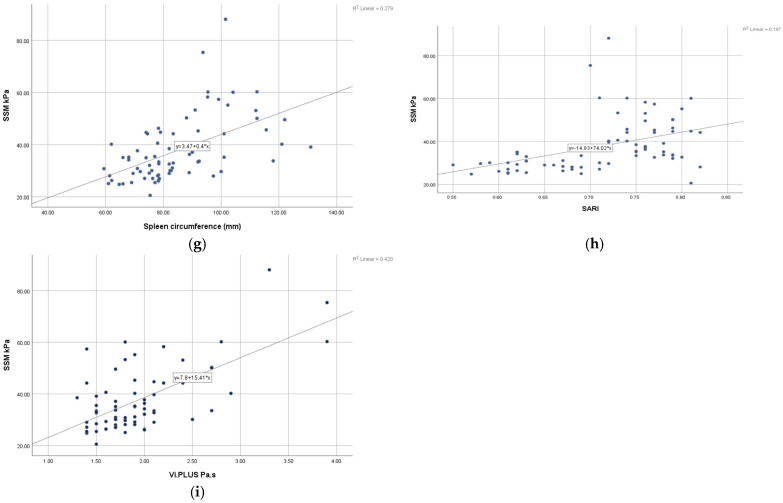
Pearson correlation analysis between (**a**) SSM and LSM; (**b**) SSM and PV diameter; (**c**) SSM and spleen size; (**d**) SSM and PVFV; (**e**) SSM and HARI; (**f**) SSM and PHI; (**g**) SSM and spleen circumference; (**h**) SSM and SARI; (**i**) SSM and ViPLUS.

The correlation diagrams show a good correlation between SSM and the ultrasound parameters, but a strong correlation index is found between SSM and LSM and also between SSM and PHI with r values greater than 0.8 (Figure 1).

**Figure 2:**
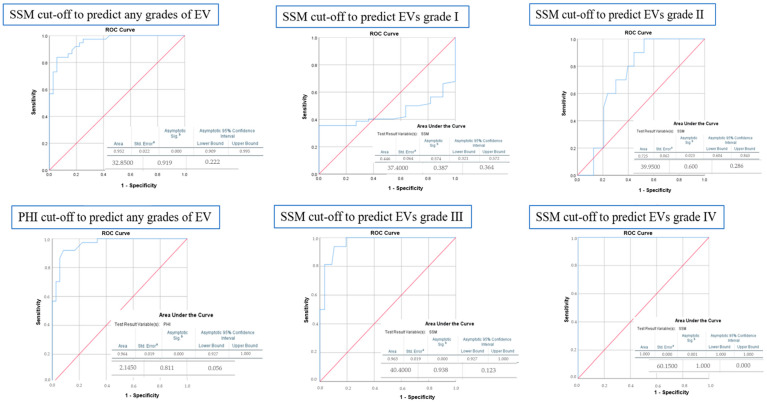
ROC curve analysis for determining the cut-off value, sensitivity, and specificity of SSM and PHI for predicting the presence or grades of EVs. ^a^ Standard Error, ^b^ Asymptotic Significance.

Figure 2 displays the cut-off values of SSM and PHI for the presence and grading of EVs. An SSM of 32.85 kPa can be highly suggestive of the presence of EVs with an AUROC of 0.95 (95% CI: 0.909–0.995, *p* < 0.001), while a gradual increase in this value cannot accurately establish the degree of EVs, although a cut-off of 40.4 kPa was associated with grade 3 EVs. Regarding PHI, a value of 2.14 can be suggestive of the presence of esophageal varices with an AUROC of 0.96.

**Figure 3:**
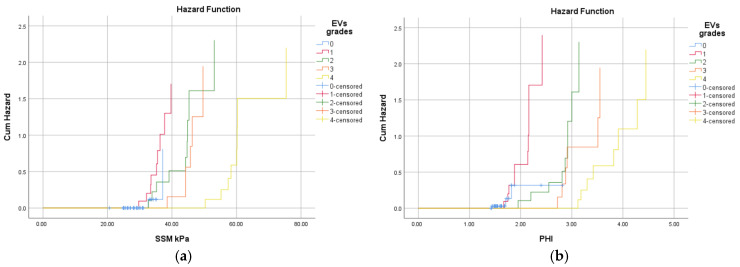
The hazard diagram plots that estimate hazard ratios (HRs) for EV grades by (**a**) SSM; (**b**) PHI.

The risk of EVs among patients with cACLD disease increases with the increase in splenic stiffness and portal hypertension index (Figure 3). Patients with SSM values greater than 32.8 kPa have a relative risk up to 3.5 times higher of having esophageal varices, and a PHI higher than 2.14 associates a relative risk of up to 1.4 times higher.

## 4. Discussion

Liver and spleen stiffness measurements have become essential non-invasive tools for evaluating patients with chronic liver disease and portal hypertension. LSM has been shown to correlate with portal hypertension and HVPG, establishing it as a key method in identifying patients at risk for complications such as esophageal varices and decompensated liver disease [19,20,21]. Furthermore, long-term studies have shown that LSM is a valuable non-invasive tool in assessing fibrosis regression following antiviral therapy in patients with chronic hepatitis C, with significant reductions in stiffness observed over a five-year period [22]. Additionally, combining SSM and LSM can enhance diagnostic accuracy and assist in patient risk stratification. There has been growing interest in the last few years regarding SSM in the evaluation of liver diseases and portal hypertension, which led to it being formally endorsed as a diagnostic tool in the 2021 Baveno VII guidelines. In the context of the Baveno VII guidelines, the use of these non-invasive measurements is increasingly relevant for reducing unnecessary endoscopies in patients with a low risk of significant esophageal varices. However, there are still limited data concerning SSM cut-off values, particularly in non-viral liver disease. This study evaluated the effectiveness of SSM by 2D-SWE as a non-invasive predictor of EVs in patients with cACLD. Our findings suggest that SSM is a valuable diagnostic tool, aligning with recent Baveno VII guidelines that advocate for non-invasive methods to stratify the risk of CSPH and reduce unnecessary endoscopies in low-risk patients [1,2].

Our results indicate a significant correlation between elevated SSM values and the presence and severity of EVs, supporting previous findings that spleen stiffness increases with portal hypertension severity [4,11,12,13,14,15,16,23,24]. Patients with SSM values above 32.8 kPa demonstrated a higher likelihood of EVs, and those exceeding 40.4 kPa were associated with more advanced variceal grades. This supports the hypothesis that spleen stiffness can serve as a surrogate marker for CSPH, reflecting increased portal pressure and variceal risk, as emphasized in the Baveno VII consensus [1]. Furthermore, our findings align with other studies demonstrating that spleen stiffness is more directly influenced by portal pressure compared to liver stiffness alone, given the spleen’s sensitivity to hemodynamic changes in the portal system [25,26,27,28,29]. More importantly, there are several studies that assessed the accuracy of SSM in predicting CSPH using the hepatic venous pressure gradient (HVPG) as the reference gold standard [30,31,32,33]. For instance, a prospective multicenter study conducted by Stefanescu et al., which aimed to evaluate SSM by using SSM@100Hz, SSM@50Hz, the presence of EV, and the HVPG, found that the best cut-off for SSM to detect HVPG ≥ 12 mm Hg was 44.95 kPa, with an AUC of 0.782 (95% CI: 0.677; 0.887) [34].

While LSM remains an essential component in non-invasive CSPH assessment, its limitations are evident, particularly in cases with indeterminate results. Approximately 40–60% of patients fall into a gray zone where LSM alone cannot accurately classify CSPH, highlighting the need for complementary measures like SSM [16]. Our study found that combining LSM with SSM improved the predictive accuracy for EV presence, consistent with reports suggesting that dual measurement may offer a more nuanced assessment of portal hypertension. Specifically, patients with SSM > 50 kPa and LSM > 25 kPa demonstrated high-risk profiles for EVs, underscoring the benefit of integrating SSM into routine evaluations for cACLD. Similarly, Dajti et al. found in a cohort of 195 patients with cACLD that CSPH was accurately ruled in in 59% of the patients when at least two of the following rule-in criteria (LSM > 25 kPa, PLT < 150 × 10^9^/L, SSM > 40 kPa) were present [35].

The implications of these findings are significant for clinical practice. Using SSM as a non-invasive tool can help tailor surveillance strategies, particularly for patients at low to intermediate risk, in whom invasive procedures like endoscopy may be deferred. This aligns with Baveno VII recommendations, which emphasize minimizing unnecessary endoscopies by using non-invasive criteria to stratify patients’ risk profiles [1]. However, there remain challenges in applying SSM universally. One limitation is the variation in SSM performance across different etiologies, as observed in cases of non-alcoholic steatohepatitis (NASH), where splenic congestion may not correlate linearly with portal pressure. Furthermore, the role of SSM across different elastography techniques (e.g., transient elastography versus point shear wave) warrants further exploration, as performance discrepancies could influence diagnostic outcomes.

Our study has several limitations. Firstly, its retrospective design may lead to selection bias and affect data collection. Additionally, the relatively small sample size may limit the generalizability of the results. To validate the established cut-off values for SSM and LSM in predicting EVs, larger studies with external validation are necessary. Although our analysis demonstrated a strong correlation between SSM and the presence of EVs, the applicability of these findings to other populations requires confirmation through prospective multicenter studies. Additionally, our exclusion criteria (e.g., splenectomy, active infection, or prior transjugular intrahepatic portosystemic shunt) may limit the applicability of the findings to broader patient populations. Future studies should aim to validate these findings across multiple centers with diverse etiologies to strengthen the evidence supporting SSM as a reliable predictor of CSPH and EVs.

## 5. Conclusions

In conclusion, SSM by 2D-SWE offers a promising, non-invasive approach to assessing the risk of esophageal varices in patients with compensated advanced chronic liver disease. By enhancing the predictive capacity for CSPH and reducing dependence on invasive procedures, SSM could play a critical role in patient stratification. However, additional studies are required to address limitations related to diagnostic thresholds, technique standardization, and etiological variations to optimize the clinical application of SSM.

## Figures and Tables

**Table 1:** Characteristics of the study population.

Patient Characteristics	*n* = 73
Age (years), median (range)	60.3 (44–76)
Sex (F/M)	16/57
BMI (kg/m^2^)	23.1 ± 5.12
**Etiology of liver disease**	
Post-hepatitis C	20 (27.4)
Alcoholic	39 (53.4)
Other etiologies	7 (9.6)
NAFLD (NASH)	7 (9.6)
Child–Pugh (A/B)	51/22
Meld-Na	8.1 ± 5.7
**Gastroesophageal varices**	
Grade 0	36 (49.3)
Grade I	11 (15.1)
Grade Il	10 (13.7)
Grade Ill	7 (9.6)
Grade IV	9 (12.3)
LSM kPa	18.58 ± 5.46
SSM kPa	37.89 ± 12.42
HARI	0.68 ± 0.71
SARI	0.71 ± 0.72
PHI	2.28 ± 0.86
Portal vein flow velocity (PVFV)	21.98 ± 6.84
Portal vein diameter (mm)	12.63 ± 1.84
Portal vein thrombosis	5 (6.8)
Splenic size (mm)	121.89 ± 16.31
Spleen circumference	85.05 ± 16.2
Splenic ViPLUS (Pa·)	1.95 ± 0.52
NSBBs	39 (53.4)

BMI, body mass index; NAFLD, non-alcoholic fatty liver disease; NASH, non-alcoholic steatohepatitis; MELD-NA, model of end-stage liver disease–sodium; LSM, liver stiffness measurements; SSM, spleen stiffness measurement; HARI, hepatic artery resistance index; SARI, splenic artery resistance index; PHI, portal hypertension index; ViPLUS, Viscosity Plane-Wave UltraSound; Pa·s, pascal second; NSBBs, non-selective betablockers.

**Table 2:** Analysis of the measured parameters according to EV grading.

Parameters	Grades of Esophageal Varices					
	0 (*n* = 36)	I (*n* = 11)	II (*n* = 10)	III (*n* = 7)	IV (*n* = 9)	*p*-Value
LSM kPa	16.81 ± 1.33	16.71 ± 2.74	18.76 ± 1.23	23.12 ± 1.11	30.02 ± 6.98	<0.001
SSM kPa	29.52 ± 3.98	35.77 ± 3.46	42.62 ± 7.35	45.51 ± 3.89	62.81 ± 11.63	<0.001
HARI	0.66 ± 0.064	0.68 ± 0.017	0.67 ± 0.60	0.74 ± 0.042	0.73 ± 0.060	0.128
SARI	0.64 ± 0.059	0.75 ± 0.26	0.78 ± 0.28	0.76 ± 0.21	0.755 ± 0.41	0.093
PHI	1.64 ± 0.27	2.09 ± 0.40	2.75 ± 0.40	3.14 ± 0.39	3.84 ± 0.67	<0.001
PVFV	26.61 ± 4.5	21.72 ± 6.34	18.6 ± 3.83	13.85 ± 3.07	13.88 ± 4.04	0.031
PV diameter (mm)	11.38 ± 1.33	14.09 ± 1.3	13.7 ± 1.88	13.14 ± 1.06	14.22 ± 2.1	0.155
Portal vein thrombosis	0	0	1	3	2	0.077
Splenic size (mm)	110.97 ± 10.68	130.54 ± 11.23	129 ± 15.62	130.14 ± 13.12	140.66 ± 13.2	<0.001
Spleen circumference (mm)	75.85 ± 8.98	85.67 ± 17.05	95.09 ± 19.91	99.24 ± 18.02	99.08 ± 7.05	0.107
Splenic ViPLUS (Pa·s)	1.70 ± 0.23	2.08 ± 0.11	2 ± 0.294	2.04 ± 0.56	2.65 ± 0.90	<0.001

LSM, liver stiffness measurements; SSM, spleen stiffness measurement; HARI, hepatic artery resistance index; SARI, splenic artery resistance index; PHI, portal hypertension index; PVFV, portal vein flow velocity; PV, portal vein; ViPLUS, Viscosity Plane-Wave UltraSound; Pa·s, pascal second.

**Table 3:** Subgroup analysis of the measured parameters.

Patient Characteristics	Evs 0 (*n* = 36)	EVs I-IV (*n* = 37)	*p*-Value
LSM kPa	16.81 ± 1.33	21.71 ± 6.25	<0.001
SSM kPa	29.52 ± 3.98	46.04 ± 12.46	<0.001
HARI	0.66 ± 0.064	0.70 ± 0.71	0.188
SARI	0.64 ± 0.059	0.76 ± 0.30	0.029
PHI	1.64 ± 0.27	2.89 ± 0.80	<0.001
PVFV	26.61 ± 4.5	17.48 ± 5.63	0.001
Portal vein diameter (mm)	11.38 ± 1.33	13.83 ± 1.42	<0.001
Splenic size (mm)	110.97 ± 10.68	132.51 ± 13.64	<0.001
Spleen circumference (mm)	75.85 ± 8.98	94.04 ± 16.68	<0.001
Splenic ViPLUS (Pa·s)	1.70 ± 0.23	2.19 ± 0.61	<0.001

LSM, liver stiffness measurements; SSM, spleen stiffness measurement; HARI, hepatic artery resistance index; SARI, splenic artery resistance index; PHI, portal hypertension index; PVFV, portal vein flow velocity; PV, portal vein; ViPLUS, Viscosity Plane-Wave UltraSound; Pa·s, pascal second.

## Data Availability

The data presented in this study are available on request from the corresponding author. The data are not publicly available because they are the property of the Institute of Gastroenterology and Hepatology, Iasi, Romania.

## References

[1] de Franchis R., Bosch J., Garcia-Tsao G., Reiberger T., Ripoll C. (2022). Baveno VII—Renewing consensus in portal hypertension. J. Hepatol..

[2] D’Amico G., Morabito A., D’Amico M., Pasta L., Malizia G., Rebora P., Valsecchi M.G. (2018). Clinical states of cirrhosis and competing risks. J. Hepatol..

[3] Ampuero J., Berzigotti A. (2023). Prognostication in Advanced Chronic Liver Disease Using Liver Stiffness Measurement: Repetita Iuvant. Gastroenterology.

[4] Ripoll C., Groszmann R., Garcia-Tsao G., Grace N., Burroughs A., Planas R., Escorsell A., Garcia-Pagan J.C., Makuch R., Patch D. (2007). Hepatic venous pressure gradient predicts clinical decompensation in patients with compensated cirrhosis. Gastroenterology.

[5] Garcia-Tsao G., Abraldes J.G. (2021). Nonselective Beta-Blockers in Compensated Cirrhosis: Preventing Variceal Hemorrhage or Preventing Decompensation?. Gastroenterology.

[6] Mendizabal M., Cançado G.G.L., Albillos A. (2024). Evolving portal hypertension through Baveno VII recommendations. Ann. Hepatol..

[7] Pennisi G., Enea M., Viganò M., Schepis F., de Ledinghen V., Berzigotti A., Wai-Sun Wong V., Fracanzani A.L., Sebastiani G., Lara-Romero C. (2023). Oesophageal varices predict complications in compensated advanced non-alcoholic fatty liver disease. JHEP Rep..

[8] Szakács Z., Erőss B., Soós A., Mátrai P., Szabó I., Pétervári E., Bajor J., Farkas N., Hegyi P., Illés A. (2019). Baveno Criteria Safely Identify Patients with Compensated Advanced Chronic Liver Disease Who Can Avoid Variceal Screening Endoscopy: A Diagnostic Test Accuracy Meta-Analysis. Front. Physiol..

[9] Karagiannakis D.S., Voulgaris T., Koureta E., Chloupi E., Papatheodoridis G.V., Vlachogiannakos J. (2019). Role of Spleen Stiffness Measurement by 2D-Shear Wave Elastography in Ruling Out the Presence of High-Risk Varices in Cirrhotic Patients. Dig. Dis. Sci..

[10] Fofiu R., Bende F., Lupuşoru R., Şirli R., Popescu A., Sporea I. (2021). Spleen Stiffness for Predicting Varices Needing Treatment: Comparison between Two Different Elastography Techniques (Point vs. 2D-SWE). Can. J. Gastroenterol. Hepatol..

[11] Reiberger T. (2022). The Value of Liver and Spleen Stiffness for Evaluation of Portal Hypertension in Compensated Cirrhosis. Hepatol. Commun..

[12] Dajti E., Ravaioli F., Zykus R., Rautou P.E., Elkrief L., Grgurevic I., Stefanescu H., Hirooka M., Fraquelli M., Rosselli M. (2023). Accuracy of spleen stiffness measurement for the diagnosis of clinically significant portal hypertension in patients with compensated advanced chronic liver disease: A systematic review and individual patient data meta-analysis. Lancet Gastroenterol. Hepatol..

[13] Wang P., Hu X., Xie F. (2023). Predictive value of liver and spleen stiffness measurement based on two-dimensional shear wave elastography for the portal vein pressure in patients with compensatory viral cirrhosis. PeerJ.

[14] Zhou H., Zhang Z., Zhang J., Sang L., Liu L., Gong X., Sun Y., Zheng Y., Yu M. (2023). Performance of spleen stiffness measurement by 2D-shear wave elastography in evaluating the presence of high-risk varices: Comparative analysis of idiopathic portal hypertension versus hepatitis B virus. BMC Med. Imaging.

[15] Karagiannakis D.S., Markakis G., Lekakis V. (2024). Evaluation of spleen stiffness by 2D shear wave elastography for ruling out high risk varices in patients with chronic advanced liver disease. A systematic review and meta-analysis. Eur. J. Radiol..

[16] Fofiu R., Bende F., Popescu A., Şirli R., Lupușoru R., Ghiuchici A.M., Sporea I. (2021). Spleen and Liver Stiffness for Predicting High-Risk Varices in Patients with Compensated Liver Cirrhosis. Ultrasound Med. Biol..

[17] Paquet K.J. (1982). Prophylactic endoscopic sclerosing treatment of the esophageal wall in varices: A prospective controlled randomized trial. Endoscopy.

[18] Sacerdoti D., Gaiani S., Buonamico P., Merkel C., Zoli M., Bolondi L., Sabbà C. (1997). Interobserver and interequipment variability of hepatic, splenic, and renal arterial Doppler resistance indices in normal subjects and patients with cirrhosis. J. Hepatol..

[19] Vizzutti F., Arena U., Romanelli R.G., Rega L., Foschi M., Colagrande S., Petrarca A., Moscarella S., Belli G., Zignego A.L. (2007). Liver stiffness measurement predicts severe portal hypertension in patients with HCV-related cirrhosis. Hepatology.

[20] Robic M.A., Procopet B., Métivier S., Péron J.M., Selves J., Vinel J.P., Bureau C. (2011). Liver stiffness accurately predicts portal hypertension related complications in patients with chronic liver disease: A prospective study. J. Hepatol..

[21] Hristov B., Andonov V., Doykov D., Doykova K., Valova S., Nacheva-Georgieva E., Uchikov P., Kostov G., Doykov M., Tilkian E. (2023). Evaluation of Liver Stiffness Measurement by Means of 2D-SWE for the Diagnosis of Esophageal Varices. Diagnostics.

[22] Facciorusso A., Del Prete V., Turco A., Buccino R.V., Nacchiero M.C., Muscatiello N. (2018). Long-term liver stiffness assessment in hepatitis C virus patients undergoing antiviral therapy: Results from a 5-year cohort study. J. Gastroenterol. Hepatol..

[23] Hu X., Huang X., Hou J., Ding L., Su C., Meng F. (2021). Diagnostic accuracy of spleen stiffness to evaluate portal hypertension and esophageal varices in chronic liver disease: A systematic review and meta-analysis. Eur. Radiol..

[24] Jachs M., Odriozola A., Turon F., Moga L., Téllez L., Fischer P., Saltini D., Kwanten W.J., Grasso M., Llop E. (2024). Spleen stiffness measurement by vibration-controlled transient elastography at 100 Hz for non-invasive predicted diagnosis of clinically significant portal hypertension in patients with compensated advanced chronic liver disease: A modelling study. Lancet Gastroenterol. Hepatol..

[25] Xu X., Liu J., Zhu Y., Rui F., Wu C., Li J. (2024). Spleen stiffness measurement as a non-invasive assessment in patients with portal hypertension. eGastroenterology.

[26] Ma X., Wang L., Wu H., Feng Y., Han X., Bu H., Zhu Q. (2016). Spleen Stiffness Is Superior to Liver Stiffness for Predicting Esophageal Varices in Chronic Liver Disease: A Meta-Analysis. PLoS ONE.

[27] Dajti E., Marasco G., Ravaioli F., Colecchia L., Ferrarese A., Festi D., Colecchia A. (2021). Risk of hepatocellular carcinoma after HCV eradication: Determining the role of portal hypertension by measuring spleen stiffness. JHEP Rep..

[28] Ravaioli F., Colecchia A., Dajti E., Marasco G., Alemanni L.V., Tamè M., Azzaroli F., Brillanti S., Mazzella G., Festi D. (2018). Spleen stiffness mirrors changes in portal hypertension after successful interferon-free therapy in chronic-hepatitis C virus patients. World J. Hepatol..

[29] Manatsathit W., Samant H., Kapur S., Ingviya T., Esmadi M., Wijarnpreecha K., McCashland T. (2018). Accuracy of liver stiffness, spleen stiffness, and LS-spleen diameter to platelet ratio score in detection of esophageal varices: Systemic review and meta-analysis. J. Gastroenterol. Hepatol..

[30] De Santis A., Nardelli S., Bassanelli C., Lupo M., Iegri C., Di Ciesco C.A., Forlino M., Farcomeni A., Riggio O. (2018). Modification of splenic stiffness on acoustic radiation force impulse parallels the variation of portal pressure induced by transjugular intrahepatic portosystemic shunt. J. Gastroenterol. Hepatol..

[31] Colecchia A., Montrone L., Scaioli E., Bacchi-Reggiani M.L., Colli A., Casazza G., Schiumerini R., Turco L., Di Biase A.R., Mazzella G. (2012). Measurement of spleen stiffness to evaluate portal hypertension and the presence of esophageal varices in patients with HCV-related cirrhosis. Gastroenterology.

[32] Elkrief L., Rautou P.E., Ronot M., Lambert S., Dioguardi Burgio M., Francoz C., Plessier A., Durand F., Valla D., Lebrec D. (2015). Prospective comparison of spleen and liver stiffness by using shear-wave and transient elastography for detection of portal hypertension in cirrhosis. Radiology.

[33] Jansen C., Bogs C., Verlinden W., Thiele M., Möller P., Görtzen J., Lehmann J., Vanwolleghem T., Vonghia L., Praktiknjo M. (2017). Shear-wave elastography of the liver and spleen identifies clinically significant portal hypertension: A prospective multicentre study. Liver Int..

[34] Stefanescu H., Marasco G., Calès P., Fraquelli M., Rosselli M., Ganne-Carriè N., de Ledinghen V., Ravaioli F., Colecchia A., Rusu C. (2020). A novel spleen-dedicated stiffness measurement by FibroScan^®^ improves the screening of high-risk oesophageal varices. Liver Int..

[35] Dajti E., Ravaioli F., Marasco G., Alemanni L.V., Colecchia L., Ferrarese A., Cusumano C., Gemini S., Vestito A., Renzulli M. (2022). A Combined Baveno VII and Spleen Stiffness Algorithm to Improve the Noninvasive Diagnosis of Clinically Significant Portal Hypertension in Patients with Compensated Advanced Chronic Liver Disease. Am. J. Gastroenterol..

